# Therapeutic Delivery of Butyrylcholinesterase by Brain-Wide Viral Gene Transfer to Mice

**DOI:** 10.3390/molecules22071145

**Published:** 2017-07-08

**Authors:** Yang Gao, Liyi Geng, Vicky Ping Chen, Stephen Brimijoin

**Affiliations:** Department of Molecular Pharmacology and Experimental Therapeutics, Kogod Center on Aging, Mayo Clinic, Rochester, MN 55905, USA; gao.yang@mayo.edu (Y.G.); geng.liyi@mayo.edu (L.G.); chen.vicky@mayo.edu (V.P.C.)

**Keywords:** butyrylcholinesterase, adeno-associated virus (AAV)-8, AAV-9, AAV-PHP.B, enzyme expression, ghrelin

## Abstract

Recent research shows that butyrylcholinesterase (BChE) is not simply a liver enzyme that detoxifies bioactive esters in food and medications. In fact, in pursuing other goals, we recently found that it has an equally important role in regulating the peptide hormone ghrelin and its impact on hunger, obesity, and emotions. Here, we present and examine means of manipulating brain BChE levels by viral gene transfer, either regionally or globally, to modulate ghrelin signaling for long-term therapeutic purposes and to set the stage for exploring the neurophysiological impact of such an intervention.

## 1. Introduction

Decades of study have brought great progress in linking the structural biology and catalytic functions of acetylcholinesterase (AChE) and, to a lesser extent, butyrylcholinesterase (BChE), two important and closely related enzymes. AChE evolved specifically to regulate and terminate neuromuscular signaling by acetylcholine, the principal neurotransmitter in brain and skeletal muscle. Its catalytic activity at synapses allows exquisite control of cognition and motor function. Its absence is lethal. A good deal of what we know today about AChE’s catalytic mechanism and biology can be credited to two Weizmann investigators: Israel Silman and Joel Sussman.

BChE inactivates acetylcholine less efficiently than AChE. It seems to have evolved in order to inactivate natural toxins in food sources, and in the modern age it appears able to protect us from chemical warfare agents and pesticides [1,2]. We, Chang-Guo Zhan, and others have used molecular modeling and mutagenesis of BChE to create a highly efficient cocaine hydrolase (CocH), to treat cocaine overdose and block drug reward for treatment-seeking addicts [3,4,5,6,7]. This “guided evolution” achieved a 1000-fold increase in catalytic efficiency, sufficient to block behavioral effects from large drug doses. In our hands, mice dosed with substantial quantities of re-engineered BChE show no reaction to a normally lethal intravenous (i.v.) administration of cocaine. 

One drawback to enzyme-based addiction therapy is the need for sustained delivery of large enzyme quantities—an expensive process. To alleviate this problem, gene transfer looks to be an ideal way forward, both for economic reasons and to provide long-sustained protection from relapse without requiring multiple treatments. A one-time gene transfer of CocH can block drug reward for several months or even longer. Thanks to the National Institute on Drug Abuse, we have now obtained preclinical CocH adeno-associated virus (AAV)-8 vector and put it into Good Laboratory Practice (GLP)-level toxicity studies in support of an FDA Investigational New Drug Permit (IND). If the results of these studies do not show ill effects, a clinical trial of gene transfer in drug users may launch within two years. Meanwhile, we are pursuing our unexpected finding that BChE is not just a “drug metabolizer” but has at least one highly specific physiological role, namely, to regulate the “hunger peptide”, ghrelin. The observations that led us to this conclusion arose in preliminary toxicity studies to determine whether sustained overexpression of BChE for addiction treatment might bring unanticipated side effects. That proved correct in a sense, but the side-effects that emerged in our study appeared to be positive rather than negative, in the sense that they led to a reduction of in-cage fighting and significantly longer life spans in male mice, which were ultimately traced to ghrelin reduction [8].

BChE’s impact on ghrelin is important because that hormone affects not only appetite and weight gain, but also emotional states, anxiety, fear, and aggression. We are now evaluating BChE delivery by viral gene transfer in mouse models as a way to modulate ghrelin signaling, hoping that such an approach to restore health and resilience might aid patients with emotional disorders or clinical obesity. With these goals in mind, several issues emerge for study. One is whether AAV8 vector treatment by peripheral i.v. injection will drive BChE expression in the brain, with unanticipated consequences that may impact cognition, memory, appetite, or emotion. To address these issues, we are conducting a range of BChE gene transfer experiments in wild-type C57BL mice and BChE knockouts (KO) engineered to eliminate expression [9]. Research is still in progress as regards behavior and cognition, but we have robust data on BChE activity across multiple brain loci in mice given viral gene transfer by different modes of injection. Here, we report on how vector type and locus impacts BChE expression in different brain loci.

## 2. Materials and Methods

Animal subjects and ethics. All tested animals were adult male C57BL/6 mice, including the BChE KO and double transgenic mice expressing a chimeric mouse/human amyloid precursor protein and a mutant human presenilin 1 (APP-PS1), and were obtained from Jackson Labs (Mayo IACUC protocol A53015). Experiments were conducted in accord with the Guide for Care and Use of Laboratory Animals [10] in a facility approved by the American Association for the Accreditation of Laboratory Animal Care.

### 2.1. I.V. Injection

cDNA encoding luciferase (Luc) or mouse BChE was subcloned into pAAV-VIP plasmid [8,11]. The Luc Or BChE plasmid was co-transfected into HEK293T cells with pHELP (Applied Viromics) and pAAV 2/8 (U. Pennsylvania) or pAAV 2/9 (U. Pennsylvania) or pAAV-PHP.B vector by introducing a 7 amino acid peptide “TLAVPFK” between amino acids 588 and 589 of VP1 gene of pAAV2/9 vector as described by Deverman et al. [12]. Virus in cell lysates was isolated by ultracentrifugation and then quantitated by real-time PCR.

### 2.2. Intracerebral Injection (I.C.)

BChE KO mice (10-weeks) were anesthetized with 50 mg/kg pentobarbital and 10 µL of AAV-BChE (1.5 × 10^12^ viral particles) was delivered as a single injection (1 µL/min), targeting the hypothalamic arcuate nucleus. Intra-cerebral injection was performed on a Kopf stereotaxic frame with mouse adapters at coordinates: A = 2.1 mm, L = 0.5 mm, V = 5.0 mm. Two-month old APP/PS1 mice were anesthetized with 50 mg/kg pentobarbital and stereotactic injection was targeted at the hippocampus and performed at coordinates: A = 2.5 mm, L =2.2 mm, V = 3.0 mm. Cannulae were left in place for 3 min and then withdrawn. The scalp incision was treated with antiseptic and antibiotics. Mice were warmed with a heating pad until they were fully conscious and mobile, after which, they were returned to their home cage, supplied with water and mouse chow, and monitored daily until fully healed.

### 2.3. Histochemistry

After euthanasia by i.p. injection of sodium pentobarbital, brains were harvested and sectioned on a Leica cryostat and transferred to glass slides for staining of BChE activity by the standard Ellman Method [13] using butyrylthiocholine as substrate. Images were captured by a low power scanning microscope.

## 3. Results

**Figure 1:**
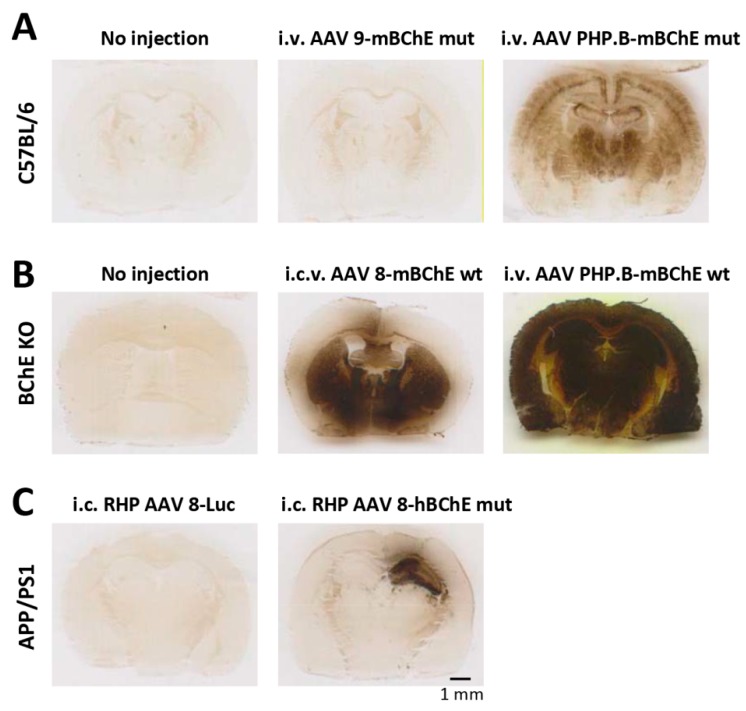
Viral gene transfer of enzyme in brains of C57BL/6, BChE KO, and APP/PS1 mice. (**A**) 2 month-old C57BL/6 mice were given i.v. injection of AAV 9 or AAV PHP.B vector coding for mouse BChE mutant at a dose of 1 × 10^13^ particles. Brains were harvested 3 weeks after injection. (**B**) 10-week-old BChE KO mice received AAV 8 vector doses of 3 × 10^12^ particles delivered stereotaxically into the intracerebroventricular (“i.c.v.”) space or tail vein i.v. injection of 1 × 10^13^ viral particles of AAV PHP.B vector coding for the same enzyme. Brains were collected 5 months or 3 weeks after injection, respectively. (**C**) APP/PS1 mice received stereotactic intracerebral (“i.c.”) injection of AAV 8-hBChE mutant or AAV 8-Luc at a dose of 3 × 10^12^ particles at 2 months of age, using coordinates for the right hippocampus (“RHP”). Six months after injection, brains were harvested for BChE activity staining.

We focused on adeno-associated viral gene transfer of BChE in brain and specifically on the issue of how vector type and route of administration would impact BChE expression in different central nervous system structures. The tested vectors were as follows: (1) AAV9 encoding mouse BChE mutated for cocaine hydrolysis and injected through tail vein; (2) AAV PHP.B, the presumptive brain-penetrating vector encoding the same mutant enzyme and the same i.v. injection route; (3) Brain sections from mice two weeks after vector or sham treatment were stained for BChE activity. In untreated KO mice, that activity was completely undetectable and in wild type mice it was not strong. However, depending on the gene transfer vector type and route of administration, brains from treated animals showed widely varied levels of BChE activity, some of them far above those seen in untreated wild-type C57BL littermate controls (Figure 1). 

Not surprisingly, mice that received BChE vector by stereotaxic injection directly into the intracerebral ventricular space showed dramatic staining in all observed brain regions except, curiously, the cerebral cortex. This structure retained low levels of activity, just slightly above those in the untreated knockout mice that had no specific staining over background (panel B).

Direct unilateral vector injection of AAV8-BChE into the hippocampus generated intense BChE staining in that structure and adjacent regions, but much less in the opposite hemisphere, which remained nearly equivalent to the untreated mice. The most dramatic result of these experiments was seen in mice that received the brain-permeating PHP-AAV vector delivered by tail vein injection. This vector led to extremely dense BChE activity staining across each section throughout the entire set of brain sections collected in this series of experiments.

## 4. Discussion

Our collective results of tests on AAV-8, AAV-9, and AAV-PHP.B vectors encoding BChE led to several interesting conclusions. First, it is abundantly clear that it is now possible to create conditions that raise BChE expression to extraordinary levels across the entire brain or in selective regions of the central nervous system. The present approaches can be used to explore the still unresolved issues of BChE’s physiological roles in the brain and elsewhere. Chief among those issues is to determine the impact of BChE, in its catalytically active form or disabled by eliminating its active-site serine, on the onset and progression of Alzheimer’s disease. A close second is to use BChE gene transfer as a means to explore the emerging issue of the enzyme’s physiologic role as a key regulator of ghrelin in general and, more particularly, in specific brain centers involved in the emotional states that are strongly influenced by this peptide hormone.

It seems worthwhile to point out that, until quite recently, AAV-9 vector was commonly viewed as the best available means of transducing neurons and other cells in the central nervous system without resorting to stereotaxic brain injections. Obviously, however, the new PHP-based vector is orders of magnitude greater in its access to brain structures once delivered into the general circulation. This viral reagent deserves strong consideration for treatments that require brain-wide loci to be targeted rather than single targets.
